# Construction of shared gene signature between rheumatoid arthritis and lung adenocarcinoma helps to predict the prognosis and tumor microenvironment of the LUAD patients

**DOI:** 10.3389/fmolb.2023.1314753

**Published:** 2024-01-10

**Authors:** Liping Shi, Houwen Zou, Jian Yi

**Affiliations:** ^1^ Department of Pharmacology, Gannan Healthcare Vocational College, Ganzhou, China; ^2^ Department of Pharmacology, Dermatology Hospital of Ganzhou, Ganzhou, China; ^3^ Department of General Surgery, First Affiliated Hospital of Gannan Medical University, Ganzhou, China

**Keywords:** rheumatoid arthritis, lung adenocarcinoma, shared signature, tumor immune microenvironment, inflammation

## Abstract

**Introduction:** Rheumatoid arthritis (RA) is a common chronic autoimmune disease with high incidence rate and high disability rate. One of the top complications is cancer, especially lung adenocarcinoma (LUAD). However, the molecular mechanisms linking RA and LUAD are still not clear. Therefore, in this study, we tried to identify the shared genetic signatures and local immune microenvironment between RA and LUAD and construct a clinical model for survival prediction.

**Methods:** We obtained gene expression profiles and clinical information of patients with RA and LUAD from GEO and TCGA datasets. We performed differential analysis and Weighted Gene Co-expression Network Analysis (WGCNA) to discover the shared genes between RA and LUAD. Then, COX regression and LASSO analysis were employed to figure out genes significantly associated with survival. qRT-PCR and Western blot were utilized to validate the expression level of candidate genes. For clinical application, we constructed a nomogram, and also explored the value of RALUADS in characterizing immune infiltration features by CIBERSORT and xCell. Finally, responses to different drug therapy were predicted according to different RALUADS.

**Results:** Our analysis identified two gene sets from differentially expressed genes and WGCNA gene modules of RA and LUAD. Filtered by survival analysis, three most significant shared genes were selected, CCN6, CDCA4 and ERLIN1, which were all upregulated in tumors and associated with poor prognosis. The three genes constituted RA and LUAD score (RALUADS). Our results demonstrated that RALUADS was higher in tumor patients and predicted poor prognosis in LUAD patients. Clinical nomogram combining RALUADS and other clinicopathological parameters had superior performance in survival prediction (AUC = 0.722). We further explored tumor immune microenvironment (TME) affected by RALUADS and observed RALUADS was closely related to the sensitivity of multiple immune blockades, chemotherapy and targeted drugs.

**Conclusion:** Our findings suggest that there are shared physiopathologic processes and molecular profiles between RA and LUAD. RALUADS represents an excellent prognosis predictor and immune-related biomarker, which can be applied to select potential effective drugs and for LUAD patients with RA.

## 1 Introduction

Rheumatoid arthritis (RA) is one type of chronic autoimmune diseases (AIDs) characterized by progressive joint degeneration and extra-auricular inflammation, leading to systemic destruction, decreased quality of life and even permanent disability (Smolen et al., 2016). According to epidemiological data, the global age-standardized prevalence rate of RA is 224.25, ranking the first in the major AIDs and demonstrated a significantly increasing trend from 1990 to 2019 (Cao et al., 2023). Although the etiology of this common AID is still uncertain, increased antibodies and immune-related substances such as rheumatoid factor and TNFα, is considered one of the most important features of RA. Therefore, drugs targeting the immune system, mainly disease-modifying antirheumatic drugs (DMARDs) is the standard treatment for RA with nanocarriers containing targeted drugs being investigated vigorously (Lin et al., 2020; Zhang et al., 2021; Smolen et al., 2023). However, in spite of the significantly improved response rate with the progression of RA treatment, increasing incidence of comorbidity and related mortality have been reported, including cardiovascular diseases (CVDs), infections, cancers etc. (England et al., 2018; Lortholary et al., 2020; Ytterberg et al., 2022; Chen et al., 2023). The underlying reasons may be systemic and chronic inflammation, genetic factors, immunosuppressant drugs and conventional risk factors such as high blood lipids level and other imbalance caused by disturbed metabolism (Szekanecz et al., 2011; Ytterberg et al., 2022).

Recent epidemiological surveys showed that the standardized incidence ratios of RA to develop cancer is 3.99 (95% CI = 3.40–4.65) (Zhou et al., 2022), with cancer types ranging from different hematological malignancies which rank the first, to other various solid tumors, such as lung cancer, melanoma, cervical cancer etc. (Zhang et al., 2022). Of note, lung adenocarcinoma (LUAD) was shown to be the most common lung cancers in AIDs (Jacob et al., 2020) and pulmonary involvement is a common extraarticular manifestation of RA (Sparks, 2019). Immune dysregulation is considered as a crucial contributor to the development of LUAD of RA patients. A bioinformatic study suggested that CD8A, GZMA, and PRF1 were related to CD8^+^ T cell in RA and positively associated with 33 tumors (Zhao et al., 2022). What’s more, MAP4K3 which interacts with and activates PKC, resulted in the activation of IKK/NF-kB in human T cells (Chuang et al., 2016). Except for T cells, Zhang etc. showed that extracellular ADP disrupted tissue homeostasis and function to trigger RA by recruiting neutrophils (Zhang et al., 2022). Nonetheless, the mechanisms under the association between RA and LUAD are still unclear yet.

Hence, in this study, we employed bioinformatic tools to demonstrate the shared mechanisms of RA and LUAD from the perspective of gene expression and clinical profiles, and immune infiltration characteristics. In order to apply to the clinical settings, we constructed a nomogram model and predict potential therapeutic drugs for LUAD, which we hope may help improve the early and effective management and treatment of LUAD.

## 2 Methods

### 2.1 Data download and processing

In order to obtain RA expression data, we applied the keyword “rheumatoid arthritis” in the Gene Expression Omnibus (GEO) dataset (GEO, https://www.ncbi.nlm.nih.gov/geo/). We search according to the following criteria: (Smolen et al., 2016): the dataset must be complete and correct, (Cao et al., 2023) samples contained in the dataset must be enough; (Lin et al., 2020) samples were collected from joint lesions rather than blood etc. Finally, we chose GSE236924, an expression profiling by array, which is normalized using RMA and contains 36 RA samples and 7 normal samples in total. The mRNA expression data of LUAD patients with corresponding clinical information were downloaded from The Cancer Genome Atlas (TCGA, https://portal.gdc.cancer.gov/) database with 541 cancer patients and 59 normal patients contained. During analysis, patients with missing information were excluded. In addition to the above two discovery cohorts, we selected another LUAD dataset, GSE229705, from GEO with complete gene expression profile data and clinical information as validation cohort, which contained paired tumor tissues and adjacent normal tissues from 123 patients. Detailed clinical information of two LUAD cohorts are shown in Supplementary Tables S1, S2.

### 2.2 Bulk gene expression data analysis

After importing the datasets and group information, we first conducted principal component analysis (PCA) of all samples in each dataset to ascertain that samples with different phenotypes demonstrate different genotypes. Then, after quality control, we used R package “Limma” and “DESeq2” to perform differential analyses for RA array dataset and LUAD RAN-seq dataset respectively. Differentially expressed genes (DEGs) were computed by setting the parameters of (|log2FC| > 1, adjusted P-value <0.05). DEG results were demonstrated as volcano plots. Further, enrichment analyses, including Kyoto Encyclopedia of Genes and Genomes (KEGG), gene ontology (GO) and Gene Set Enrichment Analysis (GSEA) were performed based on “clusterProfiler”, “org.Hs.eg.db”, “enrichplot” packages” to examine the significant pathways involved in DEGs. Finally, we identified the shared differentially expressed gene signatures via Venn gram.

### 2.3 WGCNA analysis

We used the Weighted Gene Co-Expression Network Analysis (WGCNA) tool to evaluate gene expression profiles in RA and LUAD with “WGCNA” package (Langfelder and Horvath, 2008). After removing the missing values and outliers, we constructed the co-expression networks related with clinical traits according to the best soft threshold, and chose the core modules with the highest Pearson coefficients. Hub genes in both RA and LUAD core modules were then identified and the shared gene signatures were discovered via Venn gram.

### 2.4 Construction and validation of RALUADS

For the 22 shared DEGs, we performed univariate Cox regression analysis and identified one gene with P-value <0.05. For the 62 shared WGCNA genes, we first applied the least absolute shrinkage and selection operator (LASSO) analysis for dimension reduction. After model fitting and cross validation, 29 candidate genes were finally selected according to lamda.1se. Then, we successively performed univariate and multivariate Cox regression analysis to screen out two potential genes most related with overall survival (OS). Based on the above results, we established a three-gene prognostic signature termed RA and LUAD signature (RALUADS). RALUADS for each LUAD patient is calculated by the following formula:

$$RALUADS=\sum_{i=1}^{n}Expression\left(i\right)*Coefficient\left(i\right)$$

Expression refers to the expression level of the selected gene, and Coefficient is the coefficient of the selected gene in the univariate Cox regression model. LUAD patients were divided into two groups according to the median RALUADS. Kaplan–Meier curves were drawn to assess the OS differences between different RALUADS groups. COX regression was analyzed with “survival” package and visualized with “forestplot” package. LASSO regression was performed via “glmnet” package.

### 2.5 Construction and evaluation of clinical model

We established a practical clinical model using “nomogram” package after screening out the meaningful clinical features via multivariate COX regression analysis. Then, we compared the prognostic performances of the model with other variables and in different survival time using receiver operating characteristic curve (ROC) with “timeROC” package. Additionally, calibration curve was drawn with “rms” package.

### 2.6 Pan-cancer analysis of three RALUADS genes

We used the online analysis tool TIMER 2.0 (http://timer.cistrome.org/) to systemically evaluate the differential expression level of a given gene in normal and tumor tissues across diverse cancer types in the “Diff Exp” module. The results were automatically created by the website.

### 2.7 Analysis of immune infiltration features

We used CIBERSORT (Chen et al., 2018) and xCell (Aran et al., 2017) to estimate the immune infiltration properties of TME associated with RA and LUAD. CIBERSORT is a professional analytical tool for evaluation of the abundance of different immune cells according to the reference data and gene expression profile. xCell is a novel gene signature-based *in silico* method for cell immunophenotyping with a total of sixty-four immune cells and three kinds of immune scoring systems.

### 2.8 Analysis of common drug sensitivity

The R package “oncoPredict” is specialized to predict drug sensitivity value according to the gene expression data. By calculating IC50 values, we make linear regression model to indicate the drug response efficacy to chemotherapy corresponding to different RALUADS. In order to examine the relationship between RALUADS and immunotherapy response, immunophenoscore (IPS) based on the expression of MHC molecules, markers of immunomodulators, effector cells and suppressor cells was obtained from The Cancer Immunome Atlas (https://tcia.at/) (Charoentong et al., 2017). Four types of IPS, including IPS, IPS-CTLA4 blocker, IPS-PD-1/PD-L1/PD-L2 blocker, IPS-CTLA4 & PD-1/PD-L1/PD-L2 blocker, were calculated from the TCGA-LUAD cohort.

### 2.9 Cell culture and clinical samples

Human bronchial epithelium cell line (BEAS-2B) and human lung adenocarcinoma cell lines (A549, H1975 and PC9) were purchased from Procell (Procell Life Science&Technology Co., Ltd.). All cells were cultured under standard conditions (37°C, 5% CO_2_). All cell lines were authenticated by the short tandem repeat DNA profiling test and checked for absence of *mycoplasma* contamination.

### 2.10 RNA isolation and quantitative real-time PCR (qRT-PCR)

Total RNA was extracted using TRIzol Reagent (Invitrogen, United States) according to the manufacturer’s instructions. NanoDrop was used to detect RNA concentration by A260/A280 ratio. We performed cDNA synthesis and conducted qRT-PCR with PrimeScript RT reagent kit (EZBioscience, China), and SYBR Green PCR reagent (EZBioscience, China). The reaction was incubated at 95°C for 10 min followed by 40 cycles of 95°C for 15 s and 60°C for 1 min. ACTB was used as an internal control. The primer sequences were exhibited in Supplementary Table S4. Data were analyzed using the 2^−ΔΔCT^ relative quantification method.

### 2.11 Western blot (WB) and immunohistochemistry (IHC)

To conduct WB, all cells were lysed with RIPA lysis buffer (ThermoFisher, United States) incubated on ice for 15 min. Then, the mixture was centrifuged for 2 min (12,000 × *g*, 4°C). The protein concentration of collected supernatants was measured with a BCA protein assay kit (ThermoFisher, United States). Equivalent protein was then separated by 10% Tris-Tricine SDS-PAGE and transferred onto polyvinylidene fluoride (PVDF) membranes. After being blocked in skim milk, the PVDF membranes were incubated with primary antibodies overnight at 4°C. Primary antibodies were GAPDH (Proteintech, #60004-1-Ig, RRID:AB_2107436), CCN6 (Proteintech, #21259-1-AP, RRID:AB_2918070), CDCA4 (Proteintech, #11625-1-AP, RRID: RRID:AB_2260414), ERLIN1(Proteintech, #17311-1-AP, RRID:AB_2098590). Then membranes were incubated with secondary antibody (HRP-conjugated anti-rabbit IgG, Abcam) at room temperature for 1 h. Finally, the bands on the membranes were observed with a Eestern blot substrate kit (Tanon, Shanghai, China). For IHC, we carefully searched The Human Protein Atlas, an online web tool (https://www.proteinatlas.org/), to evaluate levels of ERLIN1 in normal and tumor tissues by pathological slides.

### 2.12 Statistical analysis

All bioinformatics analyses were conducted on R software (version 4.3.1) and GraphPad Prism (Version 7.0.0). Independent sample t-tests were employed for normally distributed continuous variables to compare variables between two groups. Continuous variables that were not normally distributed were tested with Wilcoxon test. Correlation analysis between BMRGI and drug IC50 was performed with Spearman method. Statistical significance was defined with *p* < 0.05.

## 3 Results

### 3.1 Unveiling unique features of RA

**FIGURE 1:**
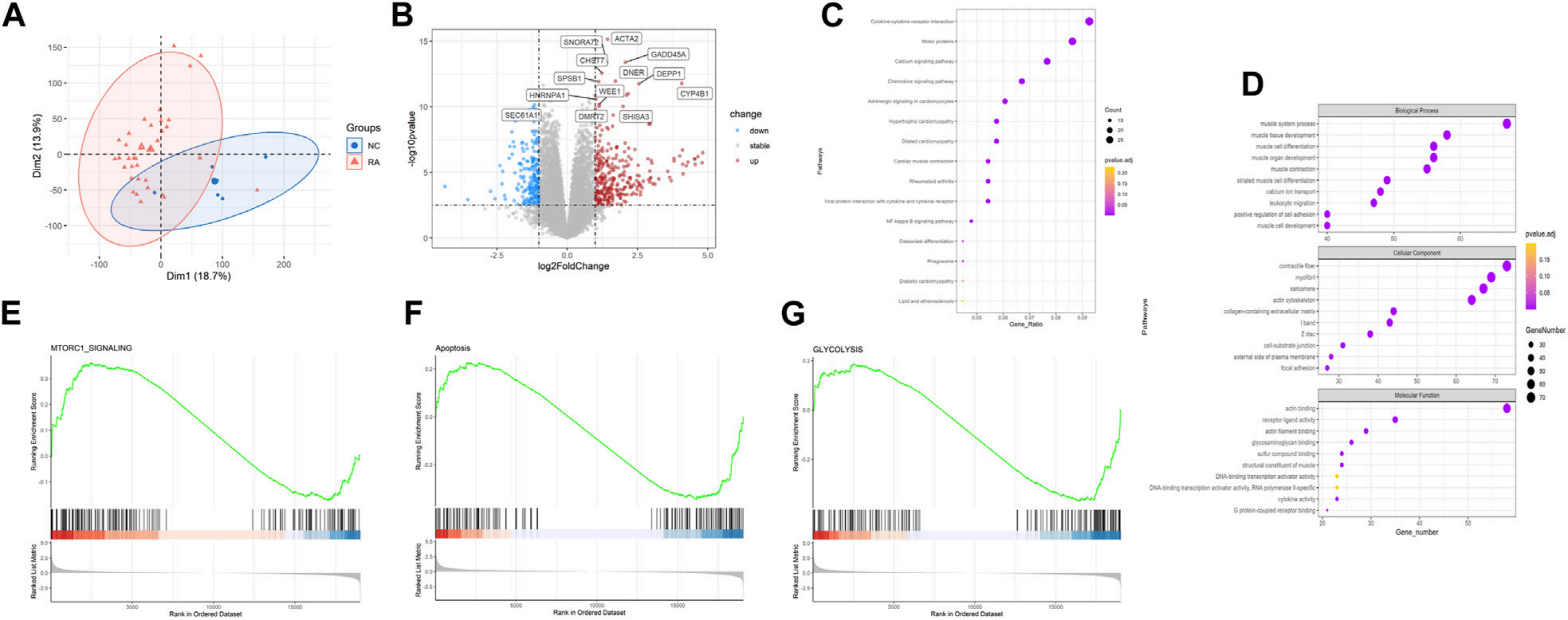
Unveiling unique features of RA. **(A)** PCA of 36 RA and 7 normal samples in GSE236924 datasets **(B)** Volcano plot of DEGs between RA and NC. **(C)** KEGG enrichment analysis of DEGs in RA compared with NC. **(D)** GO enrichment analysis of DEGs in RA compared with NC. **(E–G)** GSEA analysis of RA compared with NC. RA, rheumatoid arthritis; PCA, principal component analysis; DEGs, differentially expressed genes; KEGG, Kyoto Encyclopedia of Genes and Genomes; GO, gene ontology. Note: NC, normal tissues.

First, in order to identify genes involved in the onset and development of RA, we examined microarray profiles from 36 RA joint tissues and 7 non-RA joint tissues as control. Principle component analysis (PCA) showed distinct gene expression patterns of RA joint tissues compared with the normal ones (Figure 1A). For differential analysis (|log2FC| > 1, FDR <0.05), we identified 610 differentially expressed genes (DEGs) between RA and normal tissues (Figure 1B). Of note, CYP4B1, DEPP1, HILPDA, GADD45A and other ten genes were significantly upregulated in RA, while SEC61A1 were significantly downregulated. To reveal the functions of DEGs, KEGG analysis showed that DEGs were mainly enriched in “cytokine-cytokine receptor interaction”, “chemokine signaling pathway”, “NF-kappa B signaling pathway” and etc. Enrichment of “Rheumatoid arthritis pathway” supported that DEGs truly represented the genetic characteristics of RA (Figure 1C). The result of GO analysis showed that, in terms of Biological Process, DEGs were enriched in “muscle system process”, “muscle tissue development”, “muscle cell differentiation”, “muscle organ development”, “muscle contraction” and etc. in terms of cellular component, DEGs were enriched in “contractile fiber”, “myofibril”, “sarcomere”, “actin cytoskeleton”, “collagen-containing extracellular matrix” and etc. and in terms of molecular function, DEGs were enriched in “actin binding”, “receptor ligand activity”, “actin filament binding”, “glycosaminoglycan binding”, “sulfur compound binding” and etc., the above of which were all related to muscle and skeletal function (Figure 1D). We further conducted GSEA analysis and selected some of the most significant changed pathways to make plots. The upregulated pathway “MTORC1 SIGNALING” was reported to be associated with cell growth and further promote lung tumor progression. The downregulated pathways, “apoptosis” and “Glycolysis” might be related with cell proliferation and altered metabolism (Figures 1E–G).

### 3.2 Identification of genetic patterns of LUAD

**FIGURE 2:**
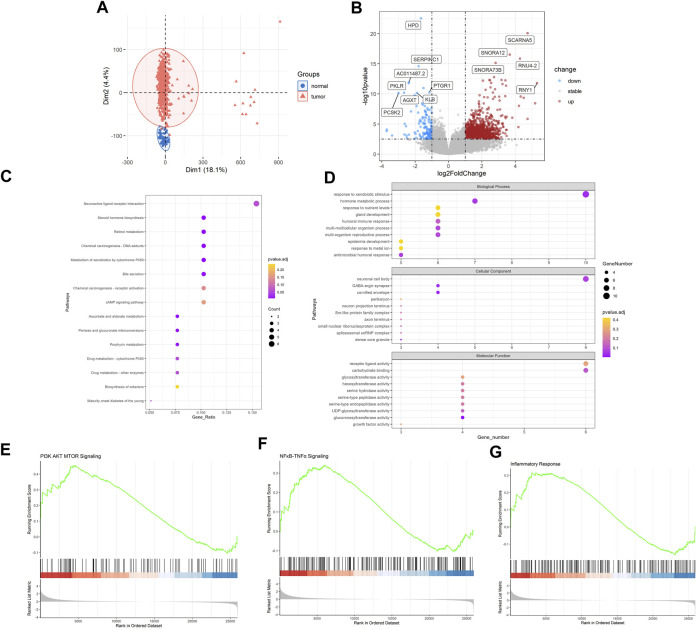
Identification of genetic patterns of LUAD. **(A)** PCA of 549 LUAD and 41 normal samples in TCGA-LUAD datasets **(B)** Volcano plot of DEGs between LUAD and NC. **(C)** KEGG enrichment analysis of DEGs in LUAD compared with NC. **(D)** GO enrichment analysis of DEGs in LUAD compared with NC. **(E–G)** GSEA analysis of LUAD compared with NC. LUAD, lung adenocarcinoma; PCA, principal component analysis; DEGs, differentially expressed genes; KEGG, Kyoto Encyclopedia of Genes and Genomes; GO, gene ontology. Note: NC, normal tissues.

Then, we sought to explore the gene expression profiles of LUAD. Also, PCA analysis evidently revealed the differential genetic patterns of LUAD *versus* normal tissue (Figure 2A). Based on differential analysis (|log2FC| > 1, FDR < 0.05), we identified 1977 DEGs (Figure 2B). Significantly upregulated DEGs in LUAD included SCARNA5, SNORA12, RNU4-2, SNORA73B, RNY1, many of which were small nuclear RNA and may be associated with post-transcriptomic splicing and modification in tumors. HPD, SERPINC1, PKLR, PTGR1, KLB, AGXT, PCSK2 were significantly downregulated. We then carried out enrichment analysis. In KEGG analysis, DEGs were mainly enriched in “neuroactive ligand-receptor interaction”, “chemical carcinogenesis-DNA adducts”, “metabolism of xenobiotics by cytochrome P450”, “cAMP signaling pathway” and etc. (Figure 2C). GO analysis demonstrated that, in terms of Biological Process, DEGs were enriched in “response to xenobiotic stimulus”, “hormone metabolic process”, “response to nutrient levels”, “gland development”, “humoral immune process” and etc. In terms of cellular component, DEGs were enriched in “neuronal cell body”, “GABA-ergic synapse”, “cornified envelope”, “perikaryon”, “neuron projection terminus” and etc. and in terms of molecular function, DEGs were enriched in “receptor ligand activity”, “carbohydrate binding”, “glycosyltransferase activity”, “hexosyltransferase binding”, “serine hydrolase activity” and etc. (Figure 2D). In addition, according to GSEA analysis, “PI3K AKT MTOR Signaling”, “NFκB-TNFα Signaling” and “Inflammatory Response” pathways were significantly upregulated (Figures 2E–G). These inflammation related pathways indicated the possible link with RA and the inflammatory tumor immune microenvironment of LUAD.

### 3.3 Analysis of shared expression networks of RA and LUAD

**FIGURE 3:**
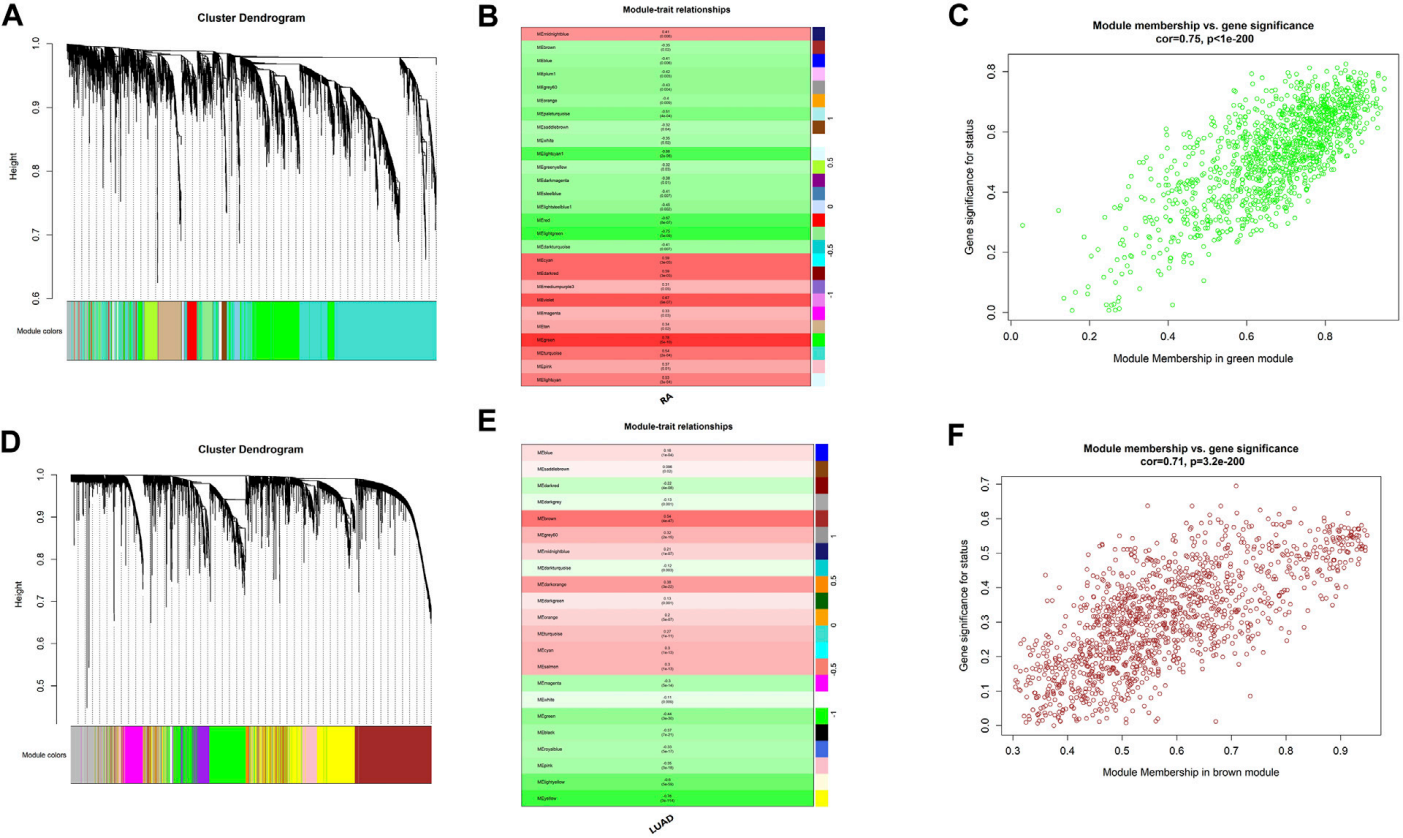
Shared expression networks of RA and LUAD. **(A,D)** Gene clustering dendrogram. **(B,E)** Correlation heatmap between modules and clinical traits. **(C,F)** Linear correlation between module membership and gene significance.

In order to explore shared molecular mechanisms of RA and LUAD more deeply, we conducted Weighted Gene Co-Expression Network Analysis (WGCNA) for the two datasets respectively according to the Section 2. For RA, by setting the soft threshold as 8 and dividing the patients into disease and normal groups (Supplementary Figures S1A), we obtained 27 modules of genes significantly related to the characteristics of RA (Figures 3A, B), of which the green module showed the highest correlation with RA (R = 0.78, P-value <0.001). Genes in the green module were highly correlated with gene significance of RA status (R = 0.75, P-value <0.001) (Figure 3C). For LUAD, the soft threshold was set as 7 and patients were divided into tumor and control groups (Supplementary Figures S1B). WGCNA results revealed that there were 22 modules significantly associated with LUAD (Figures 3C, D), and the brown module was the most significant one (R = 0.54, P-value <0.001). Also, genes in the brown module were highly correlated with gene significance of LUAD (R = 0.71, P-value <0.001) (Figure 3F).

### 3.4 Construction of the common gene signature of RA and LUAD

**FIGURE 4:**
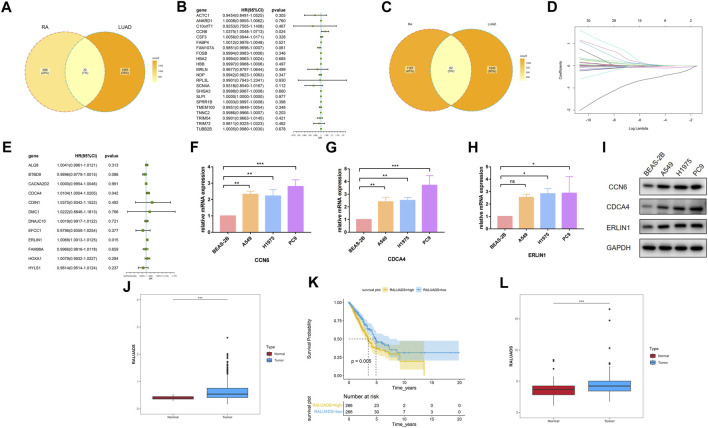
Construction of the common gene signature of RA and LUAD. **(A)** Venn plot of shared DEGs between RA and LUAD. **(B)** Forest plot showing the univariate Cox regression analysis of shared DEGs. **(C)** Venn plot of shared significant module genes between RA and LUAD. **(D)** LASSO coefficient profiles. **(E)** Forest plot showing the multivariate Cox regression analysis of selected shared significant module genes. **(F–H)** mRNA expression of CCN6, CDCA4, ERLIN1 in different cell lines by qRT-PCR. **(I)** Protein expression of CCN6, CDCA4, ERLIN1 in different cell lines by Western blot. **(J)** Expression level of RALUADS in LUAD and normal tissues in TCGA-LUAD cohort. **(K)** Kaplan-Meier survival curves stratified by RALUADS. **(L)** Expression level of RALUADS in LUAD and normal tissues in validation cohort.

Based on the findings of differential analysis and WGCNA, we tried to build a signature shared by RA and LUAD. Using Venn tool, we first showed 22 shared DEGs between RA and LUAD (Figure 4A), including ACTC1, ANKRD1, C10orf71, CCN6, CSF3, and FABP4 etc. Then, we performed univariate Cox analysis (Figure 4B) and found that only one gene, CCN6, was the candidate gene to affect overall survival (OS). For genes in two WGCNA modules, Venn plot showed 62 common genes shared by both the characteristics of RA and LUAD (Figure 4C). To select the most significant genes, we first carried out LASSO analysis for dimension reduction (Figure 4D; Supplementary Figures S1C) and found 29 genes. Then, univariate Cox analysis was performed on these genes (Supplementary Figures S1D) and 12 of them were showed to be significantly associated with survival (P-value <0.05). Further, we built multivariate Cox regression model (Figure 4E; Supplementary Table S4) and finally identified two genes, CDCA4 and ERLIN1, to be risk factors affecting OS (CDCA4: HR (95%CI) 1.0104 (1.0004–1.0205), P-value 0.042; ERLIN1: HR (95%CI) 1.0069 (1.0013–1.0125), P-value 0.015). Therefore, we concluded that CCN6, CDCA4, ERLIN1 were three candidate genes with prognostic value in LUAD patients affected by RA at the same time. The three genes all showed significantly higher expression level in tumor tissues compared with the paired normal ones in TCGA dataset (Supplementary Figures S2A–C) and demonstrated strong prognostic value that increased expression is associated with poor overall survival (Supplementary Figures S2D–F). We validated the expression level of the three genes in one normal bronchial epithelium cell line (BEAS-2B) and three human LUAD cell lines (A549, H1975 and PC9). qRT-PCR and Western blot showed that CCN6, CDCA4 and ERLIN1 were significantly upregulated in LUAD cells in terms of mRNA (Figures 4F–H) and protein (Figure 4I) levels. Pathological slides also revealed higher expression of ERLIN1 in LUAD tissues compared with normal lung tissues (Supplementary Figures S2G). Additionally, we conducted pan-cancer analysis and found that these genes had differential expression levels in various tumors compared with the normal ones (Supplementary Figures S2H–J), implying that they might play different roles in different cancers. By combining the expression value and coefficients of the selected genes according to Methods Part, we constructed RA and LUAD prognostic score, which we termed as RALUADS. RALUADS was significantly higher in patients with tumor than in normal people (Figure 4J). According to the median of RALUADS, we divided tumor patients into high RALUADS group and low RALUADS group. The kaplan-meier analysis showed that OS of high RALUADS group was significantly lower than that of low RALUADS group (Figure 4K). Another LUAD cohort from GSE229705 validated the prognostic value of RALUADS. Apart from the higher level of RALUADS in tumor tissues (Figure 4L), we conducted logistic regression and found that higher RALUADS predicted LUAD progression (OR (95%CI) 1.232 (1.0115–1.4528), P-value 0.06) and recurrence (OR (95%CI) 1.3500 (1.0882–1.6115), P-value 0.02). We further explored characteristics of patients in high RALUADS group compared with those in low RALUADS group. After differential expression analysis (|log2FC| > 1), we obtained 5389 upregulated genes and 1658 downregulated genes. KEGG analysis revealed that high RALUADS was positively related to pathways including “Systemic lupus erythematous”, “Neutrophil extracellular trap formation”, “Spliceosome”, “cAMP signaling pathway” and “MicroRNAs in Cancer” (Supplementary Figures S3). Neutrophil extracellular traps have been reported to be a bidirectional mediator of tumor progression (Adrover et al., 2023) and microRNA is a group of small non-coding RNAs which function as sponges and might exert pro-tumor effects (Alahdal and Elkord, 2023). On the other hand, high RALUADS was negatively associated with “Cellular senescence”, “p53 signaling pathway” and “Transcriptional misregulation in cancer” (Supplementary Figures S3B). Besides, GSEA analysis demonstrated multiple pathways enriched in high RALUADS group, one of which is KRAS signaling pathway, a crucial “switch” regulating cell proliferation (Supplementary Figures S3C).

### 3.5 Evaluation of the clinical value of RALUADS

**FIGURE 5:**
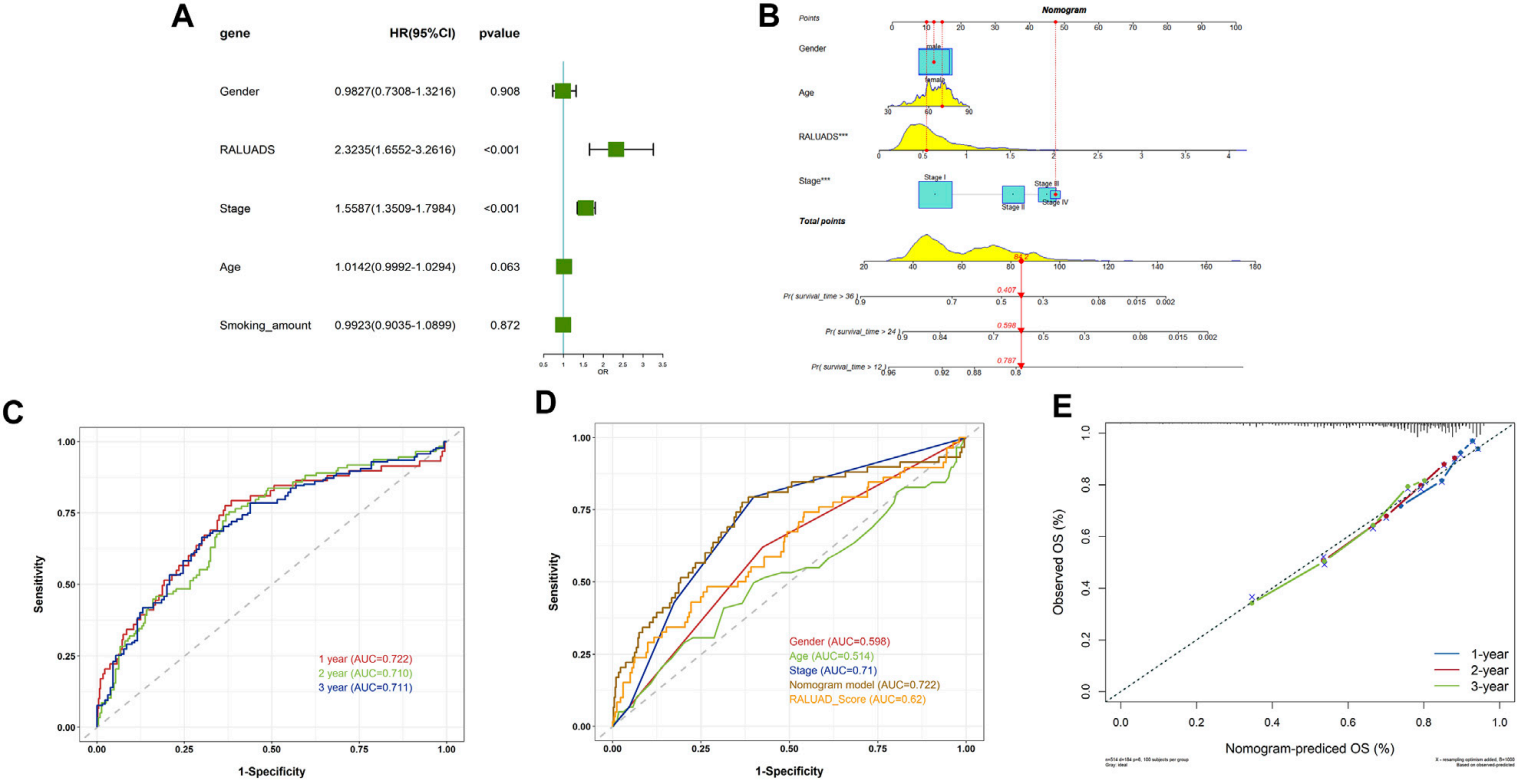
Evaluation of the clinical value of RALUADS. **(A)** Forest plot showing multivariate Cox regression analysis of common clinicopathological parameters and RALUADS. **(B)** Nomogram integrating significant clinicopathological parameters and RALUADS. **(C)** ROC curves of nomograms predicting 1-year, 2-year and 3-year survival rates. **(D)** ROC curves for clinicopathological parameters, RALUADS and nomogram. **(E)** Calibration curves for nomograms predicting 1-year, 2-year and 3-year survival rates.

In order to apply RALUADS into the clinical settings, we incorporated clinicopathological parameters, including gender, tumor stage, age and smoking amount and performed multivariate COX regression analysis. The results demonstrated that RALUADS and tumor stage were two risk factors with significant prognostic values (RALUADS: HR (95%CI) 2.3235 (1.6552–3.2616), P-value <0.001; Stage: HR (95%CI) 1.5587 (1.3509–1.7984), P-value <0.001) (Figure 5A). Although gender and age were not significant in the COX model, they were commonly considered as crucial in the development and progression in RA and LUADS. Therefore, we combined RALUADS, stage, gender and age to establish a new nomogram model to visualize the contribution of these clinicopathological parameters to OS in different years (Figure 5B). In order to evaluate the accuracy and robustness of the model, we performed ROC curve analysis and the results showed that area under the curve (AUC) of 1-year, 2-year and 3-year were respectively 0.722, 0.710 and 0.711 (Figure 5C). The prognostic performance of various clinical indices in predicting OS of LUAD was also assessed. The ROC curve revealed that compared with other traditional indicator, our nomogram model had the best performance in survival prediction (Figure 5D). In addition, we quantified the performance in terms of calibration, which showed the agreement between predictions and observations (Figure 5E).

### 3.6 Integrated analysis of correlation between RALUADS and tumor immune microenvironment in LUAD

**FIGURE 6:**
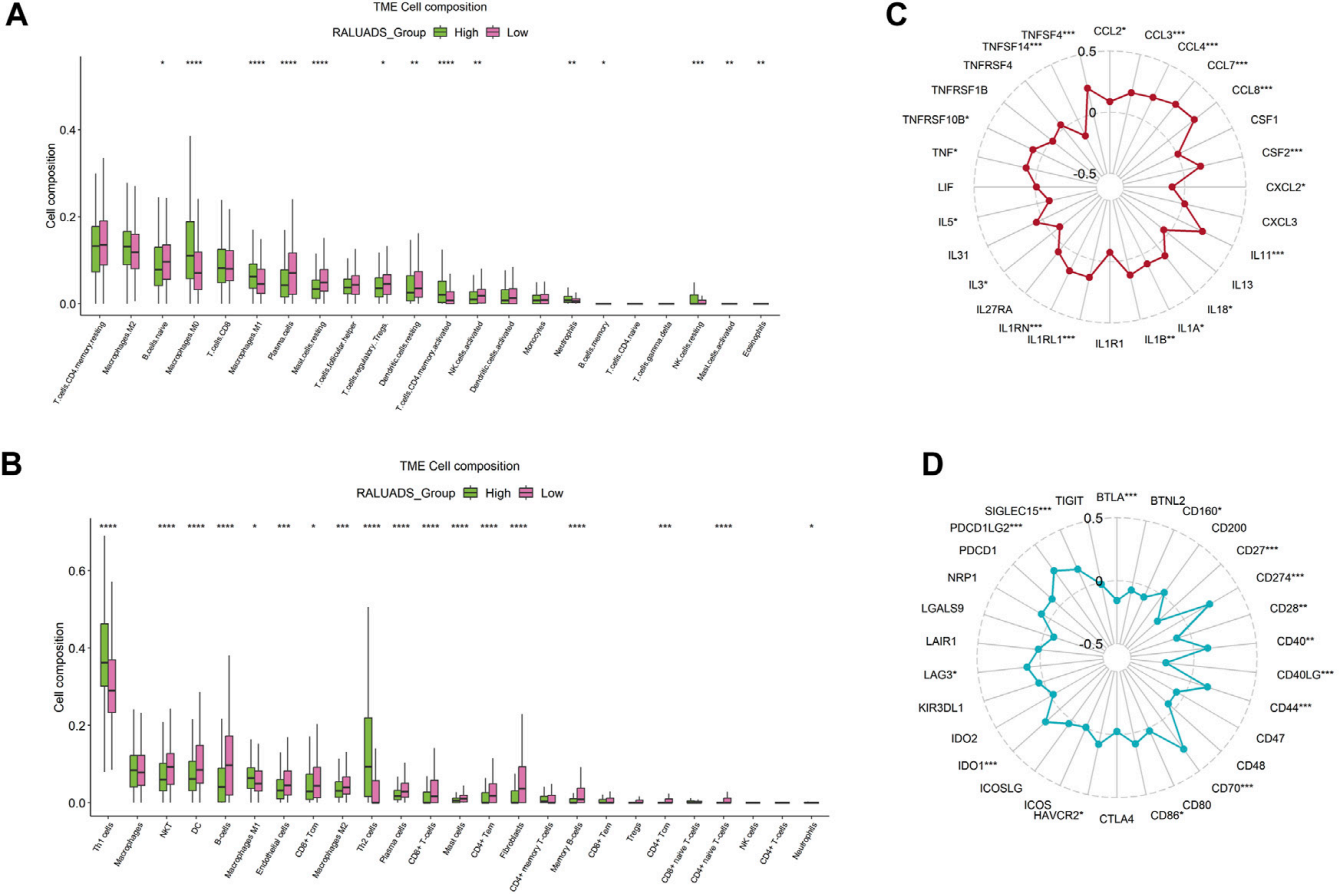
Integrated analysis of correlation between RALUADS and tumor immune microenvironment in LUAD. **(A,B)** Differences in infiltration level of various types of immune cells between high and low RALUADS groups by analysis of CIBERSORT and xCell. **(C)** Correlation between RALUADS and immune activation related molecules. **(D)** Correlation between RALUADS and immune checkpoint molecules. Note: Treg, regulatory T cell; Th1, type 1 helper T cell; DC, dendritic cell; Tcm, central memory T cell; Tem, effector memory T cell.

Tumor immune microenvironment (TME) plays a crucial role in shaping the behavior of tumor cells and interaction between tumor and stroma. Hence, we employed two analytical tools, CIBERSORT and xCell to illustrate cellular components of TME respectively. On the one hand, in the analysis of CIBERSORT, compared with low RALUADS group, the levels of M0, M1, activated memory CD4 T cells were significantly higher in high RALUADS group, while naïve B cells, plasma cells, Tregs, resting mast cells, resting dendritic cells (DCs) and activated NK cells were significantly decreased, indicating an activated TME in LUAD (Figure 6A). On the other hand, in the analysis of xCell, the levels of Th1, Th2 and M1 were significantly higher in high RALUADS group compared with the low one, while NKT cells, DCs, CD8^+^ Tcm cells, M2, plasma cells, CD8^+^ cells, CD4^+^ memory T cells, CD4^+^ Tcm cells and CD4^+^ naïve T cells were significantly decreased (Figure 6B). The two methods showed similar results in the changes of various immune cell proportions between high and low RALUADS groups. For example, the elevated level of M1 in the high RALUADS group revealed its pro-inflammatory environment and the descended level of plasma cells indicated that decreased antibody production might impair the anti-tumor ability of NK cells via antibody dependent cellular cytotoxicity. Additionally, results of xCell showed decease of a group of memory cells, implying that rapid anti-tumor response might be dampened in the lung tissue (Gebhardt et al., 2023). Then, we analyzed the levels of immune activation related molecules and immune checkpoint molecules in the TME. The radar plot showed that multiple TNF family molecules, IL family molecules and chemokines were significantly positively correlated with RALUADS (Figure 6C). What’s more, the expression level of several immune checkpoints including LAG3, HAVCR2 and PDL1 (CD274) were upregulated in high RALUADS group while BTLA, CD40LG etc. were downregulated (Figure 6D).

### 3.7 Identification of sensitive drugs in LUAD patients with RA

**FIGURE 7:**
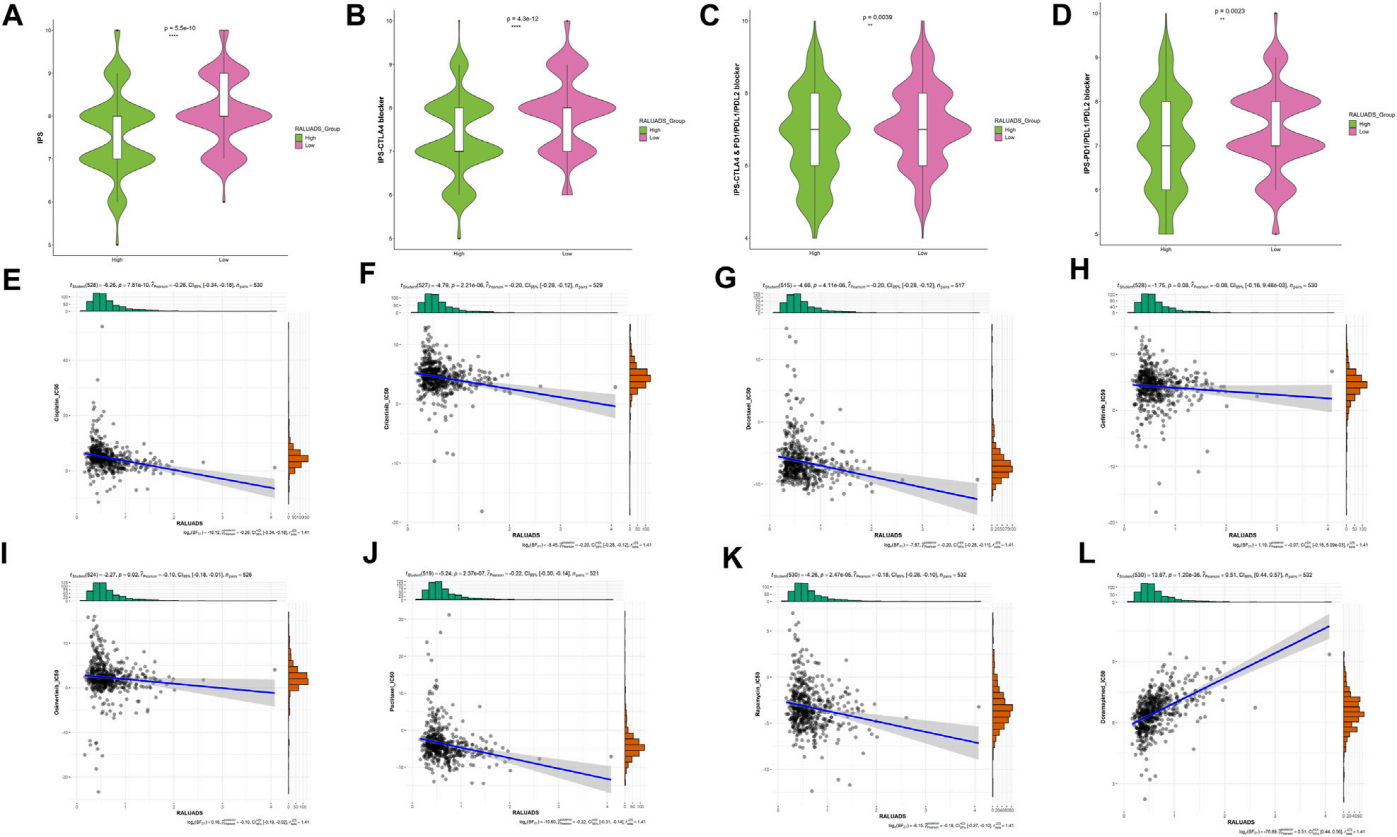
Identification of sensitive drugs in LUAD patients with RA. **(A–D)** Correlation analysis between RALUADS and response to immunotherapy. **(E–L)** Correlation analysis between IC50 value of chemotherapy or targeted drugs and RALUADS.

We finally evaluated the correlation with RALUADS and response to different therapies. Given the fact that expression level of immune checkpoints has been reported to be correlated with benefit of checkpoint blockade, we compared the immunophenoscore (IPS) and immunotherapy responses in high and low RALUADS groups. IPS was significantly higher in low RALUADS group (Figure 7A). Patients with low RALUADS could have better therapeutic effects from anti-CTLA4 (P-value <0.01), anti-PD1/PDL1/PDL2 (P-value = 0.0039) or anti-CTLA4 & PD1/PDL1/PDL2 (P-value = 0.0023) (Figures 7B–D). We next explored responses to chemotherapy drugs and targeted drugs in different RALUADS groups. A great many drugs were negatively correlated with RALUADS, indicating that patients with high RALUADS would possibly be more sensitive to Cisplatin (R2 = −0.26, P-value < 0.001), Crizotinib (R2 = −0.20, P-value < 0.001), Docetaxel (R2 = −0.20, P-value < 0.001), Gefitinib (R2 = −0.08, P-value = 0.08), Osimertinib (R2 = −0.10, P-value = 0.02), Paclitaxel (R2 = −0.22, P-value < 0.001), Rapamycin (R2 = −0.18, P-value < 0.001) (Figures 7E–K). In contrast, IC50 value of Doramapimod was positively correlated with RALUADS (R2 = 0.51, P-value < 0.001) (Figure 7L).

## 4 Discussion

AIDs are a group of diseases that usually involve the whole body with insidious clinical manifestations. RA is one of the most common chronic AIDs that presents as joint damage and extra-auricular lesions, causing heavy global burden, such as movement disability and multi-organ dysfunction. Moreover, there are studies showing the linkage between RA and different types of cancers (Szekanecz et al., 2006; Mao et al., 2020), especially lung adenocarcinoma. In recent years, the prevalence of LUAD has grown rapidly, becoming the main pathological entity in lung cancer and still presenting the low survival rate (Barta et al., 2019; Van Hal and Diab Garcia, 2021). Although there are several investigations revealing the possible mechanisms between RA and LUAD, the most distinct one is immune dysregulation. Given the fact that RA is a systemic inflammatory disease and LUAD has also its unique and heterogenous TME, we sought to explore more deeply into the shared immune mechanism between RA and LUAD. To address this issue, we first conducted differential analysis and WGCNA in RA and LUAD datasets respectively. Enrichment analysis showed that DEGs in RA participated in several tumor related pathways, such as metabolism and proliferation, while DEGs in LUAD participated in various immune processes, indicating the interaction between the microenvironment of RA and LUAD. WGCNA identified two modules significantly related to RA and LUAD. Furthermore, we subjected the overlapping genes of differential analysis and WGCNA to LASSO analysis and COX regression analysis to screen out genes that most represent prognostic value. Finally, three genes were identified as shared genes of RA and LUAD, CCN6, CDCA4 and ERLIN1, whose higher expression level in LUAD cells compared with normal epithelial cells were confirmed by qRT-PCR, WB and IHC. CCN6, CDCA4 and ERLIN1 were used to establish RALUADS. The score was significantly higher in tumor patients and correlated with poor prognosis in both the discovery cohort and validation cohort.

CCN6, or WISP3, or Wnt1‐inducible signaling pathway protein 3, belongs to CCN family and is structurally characterized by a glycosylated part and four conserved cysteine-rich domains (Holbourn et al., 2008). Members of CCN family, including CCN1‐6, are evolutionarily conserved and participate in regulating different pathophysiological processes, including cell proliferation, adhesion, angiogenesis, ECM modeling, migration, tumor growth (Jun and Lau, 2011). Studies have highlighted that expression of CCN6 is higher in RA synovium and fibroblast-like synoviocytes compared with osteoarthritis and normal synovial tissue (Cheon et al., 2004). What’s more, a recent study found that gender related lncRNA XIST could bind to GATA1, leading to CCN6 upregulation and driving RA pathogenesis by promoting SF proliferation and angiogenic activity (Yu et al., 2023). Although there is still no evidence that relates CCN6 to LUAD, CCN6 have been shown to play a crucial role in gastrointestinal cancers (Thorstensen et al., 2003), breast cancers (Djomehri et al., 2020) and chondrosarcoma metastasis to lung (Tzeng et al., 2018). CDCA4, Cell Division Cycle-Associated Protein 4, is a member of E2F family of transcription factors having a role cell cycle regulation. Pan cancer analysis revealed that a great number of tumors highly expressed CDCA4, which was associated with poor survival and different immune infiltration characteristics (Fang et al., 2022). In particular, CDCA4 was found to be a marker for poor OS in patients with LUAD (Tan et al., 2022). Although there are not evidences showing direct association between CDCA4 and RA, previous studies have shown that transcription factors in E2F family were enriched in RA (Takeshita et al., 2019). ERLIN1, also named for ER Lipid Raft Associated 1, is a part of a protein complex which mediates degradation of inositol 1,4,5-trisphosphate receptors in the endoplasmic reticulum, important for cellular cholesterol homeostasis. However, ERLIN1 functions beyond lipids metabolism. In breast cancers, ERLIN1 was discovered to be targeted by estrogen/MYC/miR-26 axis and promote cell growth (Tan et al., 2014). Moreover, in pancreatic adenocarcinoma, higher expression of ERLIN1 was correlated with poor survival and lower CD8+T cell infiltration (Chen et al., 2022). Taken together, the three genes in RALUADS were closely related to poor cancer survival. As some of them have not been reported to participate in the pathogenesis of RA or LUAD, we are the first to reveal the possible novel roles of these genes in LUAD with RA.

Based on RALUADS, we constructed a nomogram model with three other clinicopathological parameters and demonstrated that it had accurate and robust performance in predicting OS of LUAD. Furthermore, we investigated TME affected by RALUADS and found that immune infiltration was characterized by increased level of M1 and decreased levels of plasma cells in high RALUADS group. Also, immune chemotaxis and stimulation were significant in high RALUADS group while various immune checkpoints were positively or negatively correlated with RALUADS. Given the abundant and complex immune components in LUAD TME, we found that RALUADS could be a potential biomarker to predict immune infiltration and immunotherapy response. IPS tended to be higher in low RALUADS group and consistently, patients with lower RALUADS would be expected to benefit more from anti-CTLA4 and anti-PD1/PDL1/PDL2 immunotherapy. In contrast, by examining the differences in the therapeutic effects of chemotherapy and targeted medications in LUAD patients, IC50 of anticancer drugs was lower in the higher RALUADS group, including Cisplatin, Docetaxel and Gefitinib etc., indicating that patients with higher RALUADS might be more sensitive to chemotherapy and targeted drugs. Previous studies have put forward various models regarding the features and prognosis of LUAD. As extracellular matrix plays a crucial role in tumor progression and invasion, Wang et al. (2021) constructed a score with three integrin genes and metastasis-related microenvironmental pathways were enriched in high score group. What’s more, Zhang et al. (2023) established a signature with seventeen basement membrane related genes and patients with high risk had higher tumor mutation burden, lower immune score and poorer prognosis, which was consistent with our results that high RALUADS was associated with low IPS. Another study revealed the relationship of immune cell death and TME of LUAD and defined nine damage-associated molecular pattern related genes to be related to active immune activity and response to immunotherapy (Wu et al., 2023). However, a majority of these researches, focusing on LUAD, explored only one type of molecules, which might be inadequate to depict the panorama of LUAD. In comparison, we investigated the complex environment of LUAD affected by RA and screened out three genes from different gene family with diverse functions via differential gene analysis, WGCNA and survival analysis. This contributed to demonstrate the comprehensive profile of LUAD, including immune characteristics, cell death and cell metabolism etc.

**FIGURE 8:**
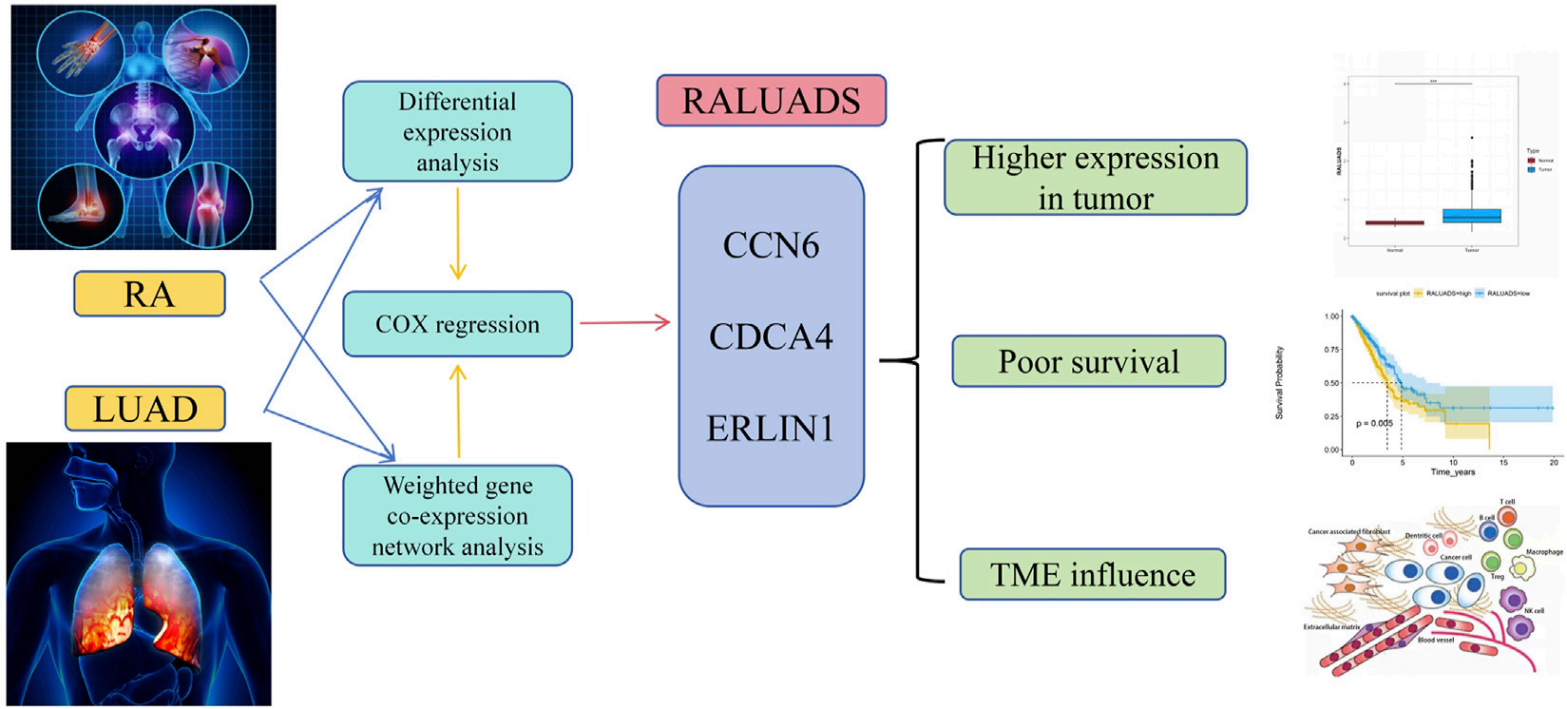
Schematic diagram of the shared signature of RA and LUAD.

In our research, we employed comprehensive analysis tools to identify the genes with the highest relationship in RA and LUAD. Three genes, CCN6, CDCA4 and ERLIN1, exhibited significant correlation and constituted RALUADS. Notably, patients with higher RALUADS showed poor prognosis, suggesting that the signature could be a promising biomarker in mortality prediction of NSCLC in MS patients. Our investigation further demonstrated the regulatory effects of RALUADS on immune infiltration and expression of immune activation molecules and immune checkpoints. In additional, RALUADS was significantly related to the effects and sensitivity of multiple therapeutic drugs. Our findings suggested that RALUADS could be applied in the clinical settings to help stratify LUAD patients and further guide the treatment strategies (Figure 8).

Our study has some limitations. First, the expression of CCN6, CDCA4 and ERLIN1 have not been validated by *in vivo* experiments. Additionally, multi-omics data may be needed to further reveal the shared characteristics of RA and LUAD. Nonetheless, our study identified RALUADS as the shared signature of RA and LUAD and provided insights of immune environment features and effective treatment therapies into the comorbidity of LUAD with RA.

## Data Availability

Datesets of discovery cohorts in this paper are available in the Gene Expression Omnibus (GEO) dataset (GEO, https://www.ncbi.nlm.nih.gov/geo/) through GSE236924 and The Cancer Genome Atlas (TCGA, https://portal.gdc.cancer.gov/) database. Dataset of validated cohort can be accessed through GSE229705. The detailed statistical data in the study are included in the article/Supplementary Material.

## References

[B1] Adrover J. M. McDowell S. A. C. He X. Y. Quail D. F. Egeblad M. (2023). NETworking with cancer: the bidirectional interplay between cancer and neutrophil extracellular traps. Cancer Cell 41 (3), 505–526. 10.1016/j.ccell.2023.02.001 36827980 PMC10280682

[B2] Alahdal M. Elkord E. (2023). Non-coding RNAs in cancer immunotherapy: predictive biomarkers and targets. Clin. Transl. Med. 13 (9), e1425. 10.1002/ctm2.1425 37735815 PMC10514379

[B3] Aran D. Hu Z. Butte A. J. (2017). xCell: digitally portraying the tissue cellular heterogeneity landscape. Genome Biol. 18 (1), 220. 10.1186/s13059-017-1349-1 29141660 PMC5688663

[B4] Barta J. A. Powell C. A. Wisnivesky J. P. (2019). Global epidemiology of lung cancer. Ann. Glob. Health 85 (1), 8. 10.5334/aogh.2419 30741509 PMC6724220

[B5] Cao F. Liu Y. C. Ni Q. Y. Chen Y. Wan C. H. Liu S. Y. (2023). Temporal trends in the prevalence of autoimmune diseases from 1990 to 2019. Autoimmun. Rev. 22 (8), 103359. 10.1016/j.autrev.2023.103359 37201621

[B6] Charoentong P. Finotello F. Angelova M. Mayer C. Efremova M. Rieder D. (2017). Pan-cancer immunogenomic analyses reveal genotype-immunophenotype relationships and predictors of response to checkpoint blockade. Cell Rep. 18 (1), 248–262. 10.1016/j.celrep.2016.12.019 28052254

[B7] Chen B. Khodadoust M. S. Liu C. L. Newman A. M. Alizadeh A. A. (2018). Profiling tumor infiltrating immune cells with CIBERSORT. Methods Mol. Biol. 1711, 243–259. 10.1007/978-1-4939-7493-1_12 29344893 PMC5895181

[B8] Chen H. Zhang J. Sun X. Wang Y. Qian Y. (2022). Mitophagy-mediated molecular subtypes depict the hallmarks of the tumour metabolism and guide precision chemotherapy in pancreatic adenocarcinoma. Front. Cell Dev. Biol. 10, 901207. 10.3389/fcell.2022.901207 35938160 PMC9353335

[B9] Chen J. Li F. Tian J. Xie X. Tang Q. Chen Y. (2023). Varicella zoster virus reactivation following COVID-19 vaccination in patients with autoimmune inflammatory rheumatic diseases: a cross-sectional Chinese study of 318 cases. J. Med. Virol. 95 (1), e28307. 10.1002/jmv.28307 36372774 PMC9878204

[B10] Cheon H. Boyle D. L. Firestein G. S. (2004). Wnt1 inducible signaling pathway protein-3 regulation and microsatellite structure in arthritis. J. Rheumatol. 31 (11), 2106–2114. 15517620

[B11] Chuang H. C. Wang X. Tan T. H. (2016). MAP4K family kinases in immunity and inflammation. Adv. Immunol. 129, 277–314. 10.1016/bs.ai.2015.09.006 26791862

[B12] Djomehri S. I. Gonzalez M. E. da Veiga Leprevost F. Tekula S. R. Chang H. Y. White M. J. (2020). Quantitative proteomic landscape of metaplastic breast carcinoma pathological subtypes and their relationship to triple-negative tumors. Nat. Commun. 11 (1), 1723. 10.1038/s41467-020-15283-z 32265444 PMC7138853

[B13] England B. R. Thiele G. M. Anderson D. R. Mikuls T. R. (2018). Increased cardiovascular risk in rheumatoid arthritis: mechanisms and implications. BMJ 361, k1036. 10.1136/bmj.k1036 29685876 PMC6889899

[B14] Fang H. Sheng S. Chen B. Wang J. Mao D. Han Y. (2022). A pan-cancer analysis of the oncogenic role of cell division cycle-associated protein 4 (CDCA4) in human tumors. Front. Immunol. 13, 826337. 10.3389/fimmu.2022.826337 35251007 PMC8891459

[B15] Gebhardt T. Park S. L. Parish I. A. (2023). Stem-like exhausted and memory CD8(+) T cells in cancer. Nat. Rev. Cancer 23 (11), 780–798. 10.1038/s41568-023-00615-0 37821656

[B16] Holbourn K. P. Acharya K. R. Perbal B. (2008). The CCN family of proteins: structure-function relationships. Trends Biochem. Sci. 33 (10), 461–473. 10.1016/j.tibs.2008.07.006 18789696 PMC2683937

[B17] Jacob S. Rahbari K. Tegtmeyer K. Zhao J. Tran S. Helenowski I. (2020). Lung cancer survival in patients with autoimmune disease. JAMA Netw. Open 3 (12), e2029917. 10.1001/jamanetworkopen.2020.29917 33315114 PMC7737093

[B18] Jun J. I. Lau L. F. (2011). Taking aim at the extracellular matrix: CCN proteins as emerging therapeutic targets. Nat. Rev. Drug Discov. 10 (12), 945–963. 10.1038/nrd3599 22129992 PMC3663145

[B19] Langfelder P. Horvath S. (2008). WGCNA: an R package for weighted correlation network analysis. BMC Bioinforma. 9, 559. 10.1186/1471-2105-9-559 PMC2631488 19114008

[B20] Lin Y. J. Anzaghe M. Schulke S. (2020). Update on the pathomechanism, diagnosis, and treatment options for rheumatoid arthritis. Cells 9 (4), 880. 10.3390/cells9040880 32260219 PMC7226834

[B21] Lortholary O. Fernandez-Ruiz M. Baddley J. W. Manuel O. Mariette X. Winthrop K. L. (2020). Infectious complications of rheumatoid arthritis and psoriatic arthritis during targeted and biological therapies: a viewpoint in 2020. Ann. Rheum. Dis. 79 (12), 1532–1543. 10.1136/annrheumdis-2020-217092 32963049

[B22] Mao J. Zhang Q. Zhang H. Zheng K. Wang R. Wang G. (2020). Risk factors for lymph node metastasis in papillary thyroid carcinoma: a systematic review and meta-analysis. Front. Endocrinol. (Lausanne). 11, 265. 10.3389/fendo.2020.00265 32477264 PMC7242632

[B23] Smolen J. S. Aletaha D. McInnes I. B. (2016). Rheumatoid arthritis. Lancet 388 (10055), 2023–2038. 10.1016/S0140-6736(16)30173-8 27156434

[B24] Smolen J. S. Landewe R. B. M. Bergstra S. A. Kerschbaumer A. Sepriano A. Aletaha D. (2023). EULAR recommendations for the management of rheumatoid arthritis with synthetic and biological disease-modifying antirheumatic drugs: 2022 update. Ann. Rheum. Dis. 82 (1), 3–18. 10.1136/ard-2022-223356 36357155

[B25] Sparks J. A. (2019). Rheumatoid arthritis. Ann. Intern Med. 170 (1), ITC1–ITC16. 10.7326/AITC201901010 30596879

[B26] Szekanecz E. Andras C. Sandor Z. Antal-Szalmas P. Szanto J. Tamasi L. (2006). Malignancies and soluble tumor antigens in rheumatic diseases. Autoimmun. Rev. 6 (1), 42–47. 10.1016/j.autrev.2006.03.007 17110316

[B27] Szekanecz Z. Szekanecz E. Bako G. Shoenfeld Y. (2011). Malignancies in autoimmune rheumatic diseases - a mini-review. Gerontology 57 (1), 3–10. 10.1159/000314634 20453490

[B28] Takeshita M. Suzuki K. Kondo Y. Morita R. Okuzono Y. Koga K. (2019). Multi-dimensional analysis identified rheumatoid arthritis-driving pathway in human T cell. Ann. Rheum. Dis. 78 (10), 1346–1356. 10.1136/annrheumdis-2018-214885 31167762 PMC6788883

[B29] Tan J. Chen F. Ouyang B. Li X. Zhang W. Gao X. (2022). CDCA4 as a novel molecular biomarker of poor prognosis in patients with lung adenocarcinoma. Front. Oncol. 12, 865756. 10.3389/fonc.2022.865756 36185189 PMC9520321

[B30] Tan S. Ding K. Li R. Zhang W. Li G. Kong X. (2014). Identification of miR-26 as a key mediator of estrogen stimulated cell proliferation by targeting CHD1, GREB1 and KPNA2. Breast Cancer Res. 16 (2), R40. 10.1186/bcr3644 24735615 PMC4053242

[B31] Thorstensen L. Holm R. Lothe R. A. Trope C. Carvalho B. Sobrinho-Simoes M. (2003). WNT-inducible signaling pathway protein 3, WISP-3, is mutated in microsatellite unstable gastrointestinal carcinomas but not in endometrial carcinomas. Gastroenterology 124 (1), 270–271. 10.1053/gast.2003.50034 12512062

[B32] Tzeng H. E. Tang C. H. Wu S. H. Chen H. T. Fong Y. C. Lu Y. C. (2018). CCN6-mediated MMP-9 activation enhances metastatic potential of human chondrosarcoma. Cell Death Dis. 9 (10), 955. 10.1038/s41419-018-1008-9 30237403 PMC6147788

[B33] Van Hal G. Diab Garcia P. (2021). Lung cancer screening: targeting the hard to reach-a review. Transl. Lung Cancer Res. 10 (5), 2309–2322. 10.21037/tlcr-20-525 34164279 PMC8182716

[B34] Wang Y. Hou K. Jin Y. Bao B. Tang S. Qi J. (2021). Lung adenocarcinoma-specific three-integrin signature contributes to poor outcomes by metastasis and immune escape pathways. J. Transl. Int. Med. 9 (4), 249–263. 10.2478/jtim-2021-0046 35136724 PMC8802404

[B35] Wu Y. Li K. Liang S. Lou X. Li Y. Xu D. (2023). An ICD-Associated DAMP Gene signature predicts survival and immunotherapy response of patients with lung adenocarcinoma. Respir. Res. 24 (1), 142. 10.1186/s12931-023-02443-0 37259066 PMC10230791

[B36] Ytterberg S. R. Bhatt D. L. Mikuls T. R. Koch G. G. Fleischmann R. Rivas J. L. (2022). Cardiovascular and cancer risk with tofacitinib in rheumatoid arthritis. N. Engl. J. Med. 386 (4), 316–326. 10.1056/NEJMoa2109927 35081280

[B37] Yu B. Chen Y. Chen E. Zuo F. Yuan Y. Zhao X. (2023). LncRNA RNA XIST binding to GATA1 contributes to rheumatoid arthritis through its effects on proliferation of synovial fibroblasts and angiogenesis via regulation of CCN6. Mol. Immunol. 153, 200–211. 10.1016/j.molimm.2022.12.004 36542956

[B38] Zhang J. Jiang L. Sun L. Wang P. Sun S. Xu M. (2021). Targeted drug delivery strategies for the treatment of rheumatoid arthritis. Sci. China Life Sci. 64 (7), 1187–1189. 10.1007/s11427-020-1920-5 34008167

[B39] Zhang X. Zhao W. Zhao Y. Zhao Z. Lv Z. Zhang Z. (2022b). Inflammatory macrophages exacerbate neutrophil-driven joint damage through ADP/P2Y(1) signaling in rheumatoid arthritis. Sci. China Life Sci. 65 (5), 953–968. 10.1007/s11427-020-1957-8 34480694

[B40] Zhang Y. Lin J. You Z. Tu H. He P. Li J. (2022a). Cancer risks in rheumatoid arthritis patients who received immunosuppressive therapies: will immunosuppressants work? Front. Immunol. 13, 1050876. 10.3389/fimmu.2022.1050876 36605209 PMC9807750

[B41] Zhang Z. Zhu H. Wang X. Lin S. Ruan C. Wang Q. (2023). A novel basement membrane-related gene signature for prognosis of lung adenocarcinomas. Comput. Biol. Med. 154, 106597. 10.1016/j.compbiomed.2023.106597 36708655

[B42] Zhao Z. Ren J. Xie S. Zou L. Zhao Q. Zeng S. (2022). Identification of biomarkers associated with CD8+ T cells in rheumatoid arthritis and their pan-cancer analysis. Front. Immunol. 13, 1044909. 10.3389/fimmu.2022.1044909 36505419 PMC9730809

[B43] Zhou Z. Liu H. Yang Y. Zhou J. Zhao L. Chen H. (2022). The five major autoimmune diseases increase the risk of cancer: epidemiological data from a large-scale cohort study in China. Cancer Commun. (Lond). 42 (5), 435–446. 10.1002/cac2.12283 35357093 PMC9118055

