# Supplementary figures and images for: Construction of shared gene signature between rheumatoid arthritis and lung adenocarcinoma helps to predict the prognosis and tumor microenvironment of the LUAD patients
Source: Front Mol Biosci. 2024 Jan 10;10:1314753. doi: 10.3389/fmolb.2023.1314753 (PMC10806137; doi:10.3389/fmolb.2023.1314753)

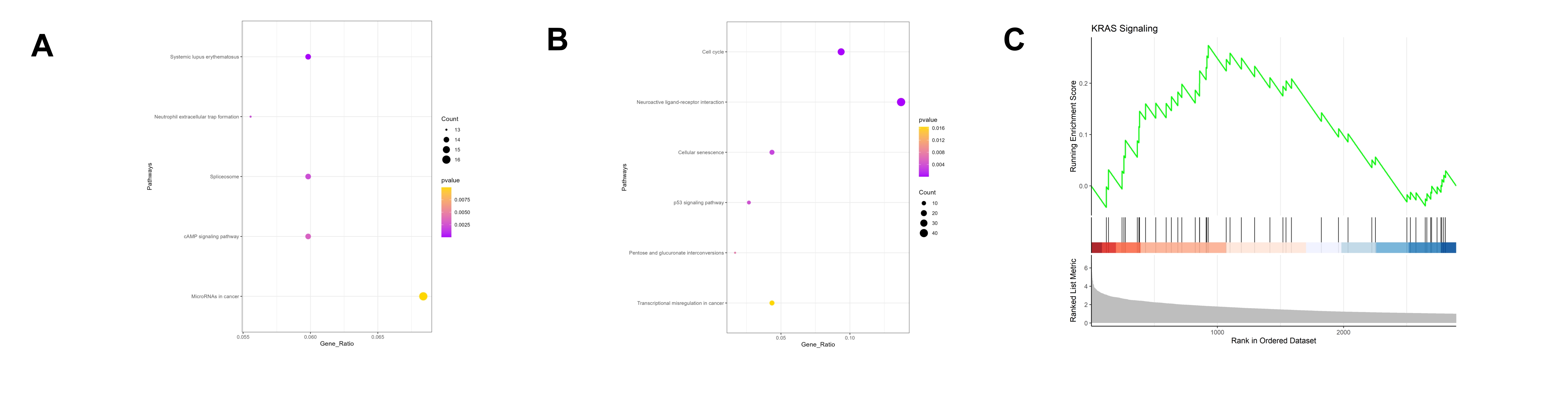

Supplement: Supplementary file 2 [file Image3.JPEG]

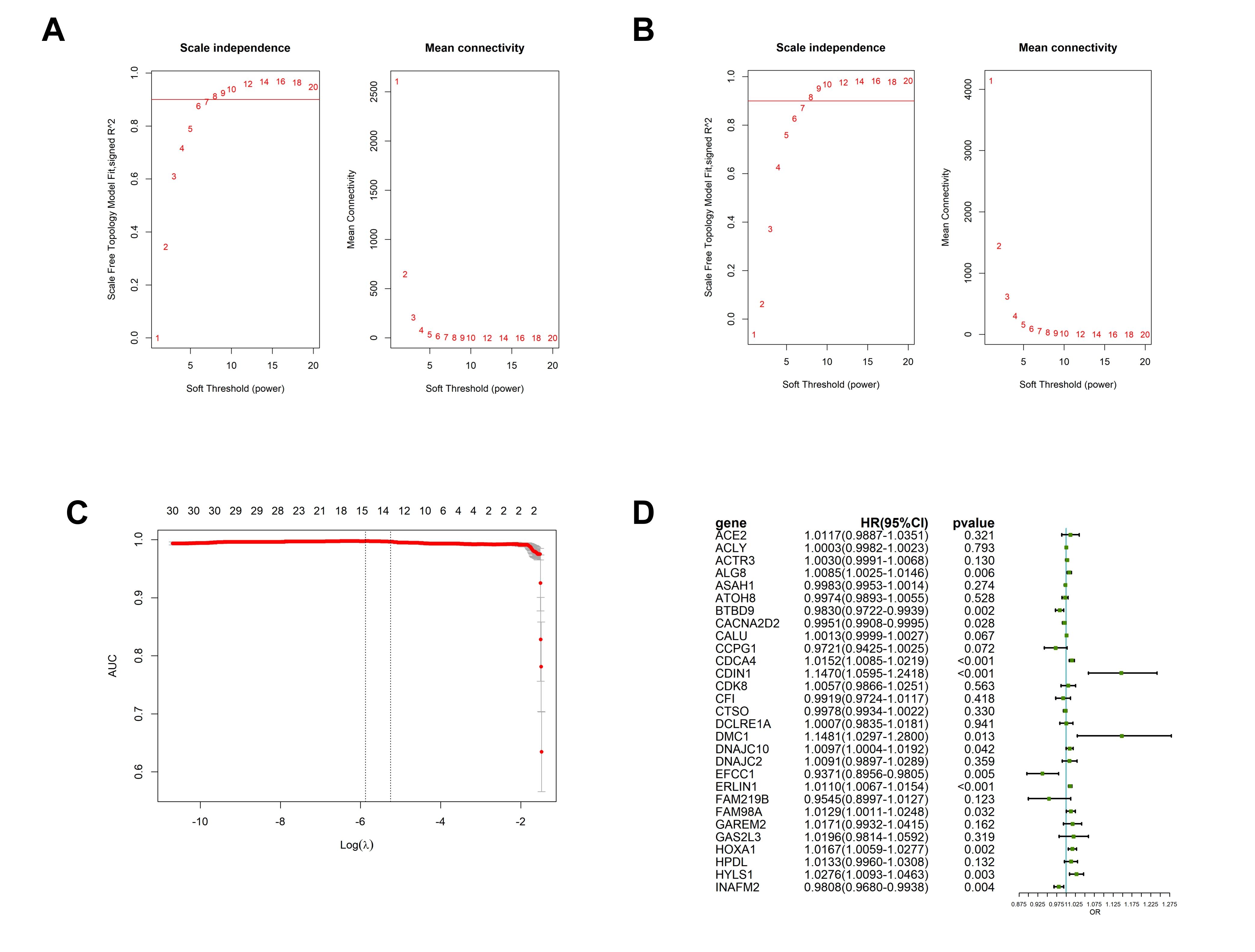

Supplement: Supplementary file 4 [file Image1.JPEG]

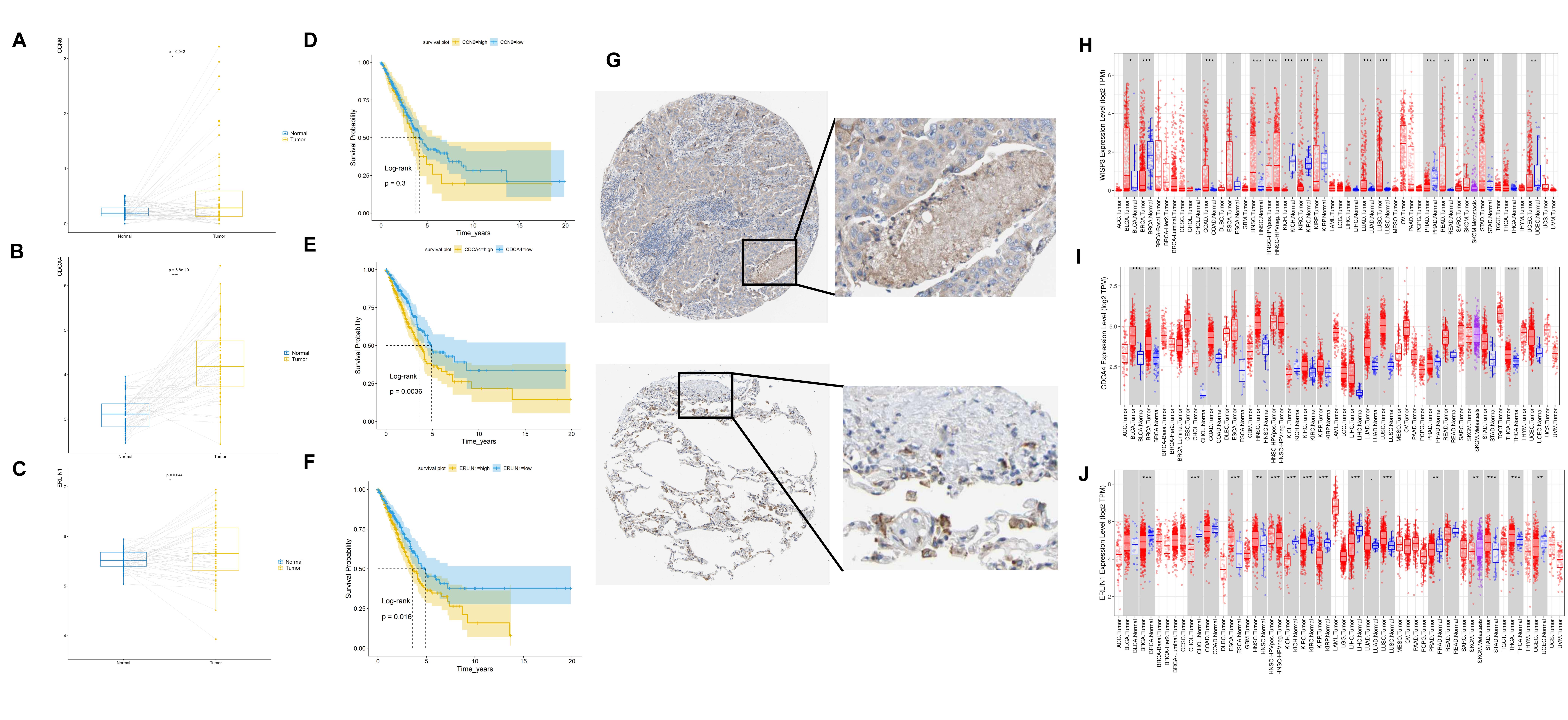

Supplement: Supplementary file 5 [file Image2.JPEG]
