# Supplementary material for: Construction of shared gene signature between rheumatoid arthritis and lung adenocarcinoma helps to predict the prognosis and tumor microenvironment of the LUAD patients
Source: Front Mol Biosci. 2024 Jan 10;10:1314753. doi: 10.3389/fmolb.2023.1314753 (PMC10806137; doi:10.3389/fmolb.2023.1314753)
Supplement: Supplementary file 8 [file DataSheet1.DOCX]

Supplementary Material

# Legend

Figure S1 Parameter selection in LASSO analysis and univariate Cox regression analysis of selected LASSO genes.

(A-B) Scale independence and mean connectivity (C) The log (lambda) sequence plot of LASSO regression. (D) Forest plot showing the univariate Cox regression results.

Figure S2 Detailed analysis of three candidate genes in RALUADS

(A-C) The expression level of CCN6, CDCA4 and ERLIN1 in paired LUAD and normal tissue. (D-F) Kaplan-Meier survival curves for LUAD patients with low or high expression of CCN6, CDCA4 and ERLIN1 stratified by median expression value. (G) The protein level of ERLIN1 on LUAD (top) and normal lung (bottom) tissue tested by IHC. (H-I) The expression of CCN6(WISP3), CDCA4 and ERLIN1 in multiple cancers and the corresponding normal tissues.

Figure S3 Comparison of characteristics of high and low RALUADS group

(A) KEGG enrichment analysis of DEGs in high RALUADS group. (B) KEGG enrichment analysis of DEGs in low RALUADS group. (C) GSEA analysis of DEGs in high RALUADS group.

Table S1 Clinical and pathological information of discovery LUAD cohort

Table S2 Clinical and pathological information of validation LUAD cohort

Table S3 Multivariate COX regression results of WGCNA genes.

Table S4 Primers for qRT-PCR.
